# The Aggregate Index of Systemic Inflammation (AISI): A Novel Prognostic Biomarker in Idiopathic Pulmonary Fibrosis

**DOI:** 10.3390/jcm10184134

**Published:** 2021-09-14

**Authors:** Angelo Zinellu, Claudia Collu, Mouhamad Nasser, Panagiotis Paliogiannis, Sabrina Mellino, Elisabetta Zinellu, Julie Traclet, Kais Ahmad, Arduino Aleksander Mangoni, Ciriaco Carru, Pietro Pirina, Alessandro Giuseppe Fois, Vincent Cottin

**Affiliations:** 1 Department of Biomedical Sciences, University of Sassari, 07100 Sassari, Italy; panospaliogiannis@gmail.com (P.P.); sabrinamellino3@gmail.com (S.M.); carru@uniss.it (C.C.); 2 Department of Clinical, Surgical and Experimental Medicine, University of Sassari, 07100 Sassari, Italy; claudia_collu@hotmail.it (C.C.); pirina@uniss.it (P.P.); agfois@uniss.it (A.G.F.); 3 Department of Respiratory Medicine, National Coordinating Reference Centre for Rare Pulmonary Diseases, Louis Pradel Hospital, 69677 Lyon, France; mouhamad.nasser@chu-lyon.fr (M.N.); julie.traclet@chu-lyon.fr (J.T.); kais.ahmad@chu-lyon.fr (K.A.); vincent.cottin@chu-lyon.fr (V.C.); 4 Unit of Respiratory Diseases, University Hospital Sassari (AOU), 07100 Sassari, Italy; elisabetta.zinellu@aousassari.it; 5 Discipline of Clinical Pharmacology, College of Medicine and Public Health, Flinders University, Bedford Park, SA 5042, Australia; arduino.mangoni@flinders.edu.au; 6 Claude Bernard University Lyon 1, UMR754, IVPC, 69007 Lyon, France

**Keywords:** AISI, complete blood cell count, derived blood cell count, idiopathic pulmonary fibrosis

## Abstract

Variable patterns of disease progression are typically observed in patients with idiopathic pulmonary fibrosis (IPF). We sought to determine the prognostic capacity of blood cell count indexes, derived from routine complete blood cell (CBC) count, in a cohort of IPF patients. The neutrophil-to-lymphocyte ratio (NLR), derived neutrophil-to-lymphocyte ratio (dNLR), monocyte-to-lymphocyte ratio (MLR), platelet-to-lymphocyte ratio (PLR), systemic inflammation index (SII), systemic inflammation response index (SIRI), and aggregate index of systemic inflammation (AISI) were calculated at baseline in a consecutive series of 82 IPF patients followed for four years. After adjusting for age, gender, body mass index, smoking status, and disease stage, only the AISI was significantly associated with mortality (HR 1.0013, 95% CI 1.0003–1.0023, *p* = 0.015). Patients with AISI <434 and ≥434 had a median survival from the diagnosis of 35.3 ± 15.2 and 26.6 ± 16.3 months (*p* = 0.015), and a four-year survival rate of 54% and 34%, respectively. The AISI, easily derivable from routine laboratory tests, is independently associated with mortality in patients with IPF. Prospective studies in larger cohorts are required to confirm this association.

## 1. Background

Idiopathic pulmonary fibrosis (IPF) is a chronic interstitial lung disease of unknown aetiology [1], characterized by a radiological and histological pattern of usual interstitial pneumonia (UIP) with an excessive deposition of extracellular matrix components, including collagen, that leads to parenchymal fibrosis [2,3]. IPF mainly affects ex-smoker adults over 65 years and has a poor prognosis, with a median survival of 3–5 years following diagnosis. It is characterized by dyspnoea, dry cough and a progressive decline in lung function and quality of life in most patients [1,4,5]. The antifibrotic drugs pirfenidone and nintedanib, approved for IPF treatment, despite being more effective than previous therapies at reducing lung function decline, are ineffective in improving lung function or preventing the progression of the disease. The latter can be difficult to predict as some patients may remain stable for long periods, whereas others show a stepwise progression pattern or a rapid symptom deterioration. To discriminate patients with different prognoses, several pulmonary function parameters have been proposed, as well as scores including clinical, functional, radiological, and haematological data [6,7,8,9,10]. Risk stratification is crucial for optimizing disease management, including scheduling for lung transplantation. Therefore, there is an intensive focus on the identification of new biomarkers with improved predictive performance. In this context, several blood-cell-derived inflammation indexes have gained an increasing interest over the last few years. Although it remains unclear whether inflammation is the cause or the effect of the fibrotic process, recent studies have shown that inflammatory cells are involved in several steps of IPF pathogenesis [11]. The complete blood cell count is routinely performed and relatively inexpensive. Combined haematological indexes of inflammation, particularly the neutrophil-to-lymphocyte ratio (NLR), derived neutrophil-to-lymphocyte ratio (dNLR), monocyte-to-lymphocyte ratio (MLR), platelet-to-lymphocyte ratio (PLR), systemic inflammation index (SII), systemic inflammation response index (SIRI), and aggregate inflammation systemic index (AISI) are increasingly studied as biomarkers in several disorders [12,13,14,15,16] including respiratory pathologies such as COPD (chronic obstructive pulmonary disease), asthma [17,18,19], and more recently, IPF [20,21]. However, no information is currently available regarding their ability to predict mortality in the latter group. We sought to determine the predictive capacity of the NLR, dNLR, PLR, LMR, SII, SIRI, and AISI in a well-defined cohort of patients with IPF.

## 2. Methods

We retrospectively studied 82 patients diagnosed with IPF at the National Reference Center for Rare Pulmonary Diseases in Lyon, France between January 2006 and July 2015. All patients were followed up for a period of four years, with no censoring. This period was longer than the conventional follow up, three years, in IPF studies. IPF was diagnosed according to the 2011 American Thoracic Society/European Respiratory Society (ATS/ERS) guidelines. Patients diagnosed before 2011 also met the criteria of these guidelines following a consensus by a multidisciplinary team that included respiratory, pathology, and radiology experts in interstitial lung disease [1]. Lung function parameters were assessed according to the criteria published by the ATS and the ERS [22].

Age, gender, body mass index (BMI), respiratory function tests, complete cell blood counts, pharmacological treatment (steroids, antiplatelet drugs, and anticoagulants), and disease stage at diagnosis were retrieved from clinical records and stored in a database. Telephone interviews were conducted to ascertain survival status when the latter was not available from records. Exclusion criteria included malignancy, bleeding tendency, severe hepatic or renal disease, lung transplantation, the ongoing use of antifibrotic drugs before the study, and use of immunosuppressants, interferon, D-penicillamine, colchicine, or oral corticosteroids during the preceding three months. The study was approved by the Institutional Ethical Committee of the Hospital Louis Pradel, Lyon, France, and the Institutional Local Ethics Committee of the University Hospital (AOU) of Cagliari, Italy (PG/2018/4426).

The derivative blood cell count inflammation indexes included the neutrophil-to-lymphocyte ratio (NLR: neutrophils/lymphocytes), neutrophil-to-lymphocyte ratio [dNLR: (WBC-lymphocytes)/lymphocytes)], monocyte-to-lymphocyte ratio (MLR: monocytes/lymphocytes), platelet-to-lymphocyte ratio (PLR: platelet/lymphocyte), systemic inflammatory index (SII: neutrophils × platelets/lymphocytes)], systemic inflammatory response index (SIRI: neutrophils × monocytes/lymphocytes), and aggregate index of systemic inflammation (AISI: neutrophils × platelets x monocytes/lymphocytes).

Results were expressed as mean values and standard deviation (SD), or median values and interquartile ranges (IQR). Individual variable distribution was assessed by the Shapiro–Wilk test.

Student’s t-test or a Mann–Whitney rank sum test were used to assess between-group differences. Correlations between variables were estimated using Spearman’s or Pearson’s correlation, as appropriate. For survival analysis, time zero was defined as the time of diagnosis. Survival probability was estimated using the Kaplan–Meier method and the log-rank test, with death being the end point. Cox proportional hazards regression was performed for both univariate and multivariate analyses, with specific focus on the independent effect of AISI on survival by controlling for potential confounders, i.e., age, gender, BMI, smoking status, disease stage, and therapy. Hazard ratios were calculated from the Cox analysis. A *p*-value < 0.05 was considered statistically significant. Statistical analyses were performed using MedCalc for Windows, version 15.4 64 bit (MedCalc Software, Ostend, Belgium).

## 3. Results

One-hundred and ten consecutive patients with IPF were initially screened. Among them, 28 were excluded because of missing data or lung transplantation. Therefore, 82 cases were enrolled in the study. Baseline characteristics are described in Table 1. The mean age at the time of diagnosis was 72 ± 7 years and 73 (89%) were males. The mean BMI was 26.8 ± 4.0 kg/m^2^, and the cohort was mainly composed of ex-smokers (74%). During the follow up, 56% of patients were commenced on pirfenidone and 8% on nintedanib. At the end of the 48-month follow up, 45 patients (55%) died. In a univariate correlation analysis, the simple and combined blood cell count indexes were significantly associated with functional lung parameters (Table 2 and Table 3).

**Figure 1:**
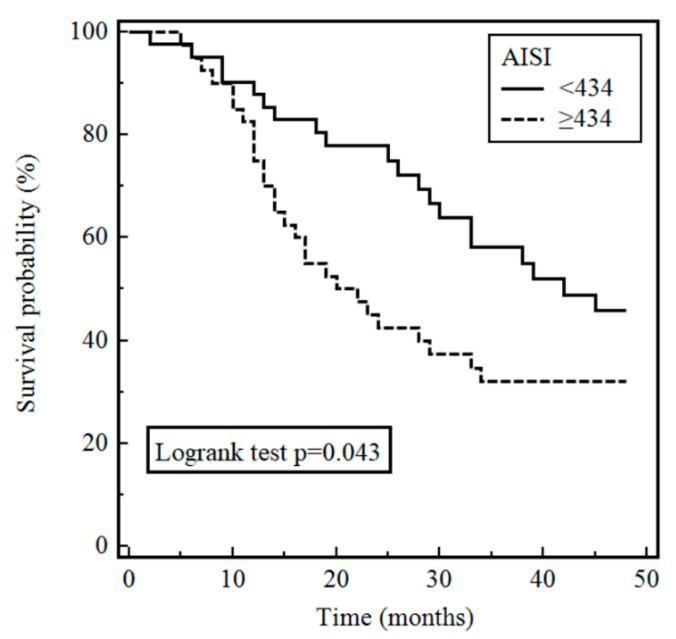
Survival of patients with idiopathic pulmonary fibrosis stratified by AISI at baseline.

Kaplan–Meier survival analysis was conducted to analyse potential prognostic factors (Table 4). The log-rank tests were performed comparing two groups defined on the basis of the median value of variable distributions. Our tests revealed that an AISI ≥ 434 was significantly associated with death (*p* = 0.043), whereas no significant associations were observed with other blood cell count indexes. There was a significant difference in survival between the two groups stratified on the basis of AISI values (group 1, patients with an AISI < 434 and group 2, patients with an AISI ≥ 434). Patients with an AISI < 434 and ≥ 434 had a median survival from the diagnosis of 35.3 ± 15.2 and 26.6 ± 16.3 months (*p* = 0.015), and a four-year survival rate of 54% and 34%, respectively (Figure 1).

The Cox proportional hazards regression showed that the risk of mortality was significantly associated with AISI values, even after adjusting for age, gender, BMI, smoking status, antifibrotic drugs treatment, and disease stage (HR 1.0017, 95% CI 1.0006–1.0027, *p* = 0.015) (Table 5). Of all the covariates, only the BMI was associated with mortality (HR = 0.8589; 95% CI, 0.7685–0.9601, *p* = 0.007), whereas neither age, gender, smoking status, antifibrotic drugs treatment, or disease stage at baseline were significantly associated with survival in the adjusted model (*p* = 0.16, 0.76, 0.14, 0.97 and 0.36, respectively).

Table 6 demonstrates the demographic, clinical, and haematological characteristics of IPF patients stratified according to AISI values. FEV_1_%, FVC% and 6MWT were significantly lower in patients with an AISI ≥ 434 compared with the group with AISI values < 434. A non-significant trend toward a decrease in TLC% in patients with an AISI ≥ 434 was also observed. There were no significant between-group differences in age, gender, BMI, smoking status, and disease stage. WBC, monocytes, neutrophils, and platelets were significantly higher in patients with an AISI ≥ 434 compared with the group with AISI values < 434, whereas lymphocytes were lower in patients with an AISI ≥ 434.

## 4. Discussion

Given the established relationship between IPF condition and some inflammation biomarkers [20,21,23,24,25,26,27,28], we sought to determine the prognostic capacity of the combined blood cell count indexes, a group of well-recognized biomarkers of inflammation obtained by the complete blood cell count test, that is routinely provided in clinical practice. Ratios of blood cell counts have shown to be effective in predicting the survival of those with acute and chronic diseases [29,30,31,32,33,34]. Similar properties have also been exhibited by some blood cell dimensional indexes, such as the red blood cell distribution width (RDW), which has been reported to be an indicator of poor prognosis in IPF [10,35]. The values of combined blood cell count indexes in the present study were similar to those recently described in IPF patients [20,21]. In addition, by univariate correlation analysis, we found significant relationships between ratios of blood cell counts and lung functional parameters FEV1%, FVC% and DLCO%, indicating that the deterioration in lung function may be related to a higher inflammatory state in IPF patients. These data are consistent with previous observations in which a significant inverse relation was observed between lung functional parameters FVC% or DLCO% and the inflammation biomarkers IL-4, IL-8 and ICAM-2 [25,26,27]. Similarly, we found that the 6MWT performance was inversely related to all combined inflammation haematological indexes measured, indicating that a pro-inflammatory state was also related to a reduced endurance exercise capacity. This finding is consistent with previous data observed in IPF patients, indicating a negative correlation between 6MWT and calgranulin B, a small calcium-binding protein, which is expressed by circulating neutrophils and monocytes, with several immunological functions [36]. Only with the SIRI did we find a significant positive association with disease severity at baseline, whereas SII, SIRI and AISI were inversely related to survival time by univariate correlation analysis. However, only the AISI was significantly associated with poor prognosis by the Kaplan–Meier survival analysis. This finding might have a significant clinical relevance in the context of mortality risk assessment in IPF patients. Stratification of disease severity is usually based on physiologic parameters, primarily baseline and/or temporal change in FVC % and or DLCO %. The absence of a valuable circulating biomarker that allows the stratification of risk has mandated the search for novel outcome predictors. In this study, we have demonstrated the prognostic utility of the AISI, a simple and routinely derived biomarker.

Although the pathophysiology of IPF remains to be fully elucidated, several immune cells are likely to be involved, particularly lymphocytes, neutrophils, and monocytes. Alterations in lymphocytes and neutrophils levels have previously been reported in IPF [20,21], as well as the association between increased monocytes values and poor prognosis [37]. Therefore, a predictive tool such as the AISI, that includes lymphocytes, neutrophils, platelets, and monocytes, might be superior to simpler indexes as it better reflects the inflammatory status in the context of specific disease states. This proposition is supported by other reports on the association between the AISI and hospital stay in open elective thoracic surgery [12], age-related macular degeneration [15], IPF [21], and COVID-19 [38]. The significant association between AISI and mortality remained after correction for potential confounding factors, including age, gender, smoking status, antifibrotic drug treatment, disease stage, and BMI (that, at higher values, was instead related to a more favourable prognosis). Due to the limited sample size, we only included age, gender, smoking status, antifibrotic drugs treatment, BMI, and disease stage in our multivariate analysis. Increasing the number of variables in the model would have reduced the statistical power, thus increasing the risk of a type 1 error. We opted to include, in the multivariate model, the disease stage instead of the lung functional parameters, since the former is calculated through the GAP index formula that integrates both FVC% and DLCO% parameters.

A multivariate Cox analysis also showed a significant inverse association between BMI and mortality. Associations between BMI and outcomes have been reported in patients with COPD [39,40]. Similar relationships between low BMI and poor prognosis have been described in IPF [41,42], although other studies have failed to confirm this association [43]. Recently, it has also been reported that the incorporation of BMI in an adimensional index that includes FVC%, DLCO%, and 6MWD has a superior capacity to predict mortality in IPF, when compared to an index that only includes FVC%, DLCO%, and 6MWD [44].

Moreover, the multivariate analysis failed to identify significant associations between smoking status, gender, age, and survival. There are conflicting results in the literature regarding the link between smoking status and survival. Several studies have shown a high percentage of smokers among patients with IPF [45,46,47], and that cigarette smoking is an independent risk factor for the development of IPF [48] and a poor prognosis [49,50]. Conversely, other studies have either failed to identify significant associations between smoking status and prognosis [44], or reported increased survival in IPF patients who were cigarette smokers at baseline when compared with former smokers or non-smokers [51,52]. Another point worth of discussion is the impact of sex. Our patients were predominantly men, in agreement with previous studies reporting a proportion of males between 67% and 77% in IPF [53,54,55] as well as a predominance of males, 78–82%, in intervention trials [56,57,58]. In agreement with recent observations [44,59], survival was not significantly associated with sex; however, this is in contrast with other studies in which the female sex was significantly associated with a lower risk of in-hospital mortality [60,61]. Moreover, in contrast with other reports [62,63], age was not independently associated with survival. However, it should also be highlighted that this observation is in line with other studies [64,65]. Song et al. [65] have suggested that IPF is predominantly a disease of older men, where increasing age and the male sex predict IPF onset, but less so survival. Conflicting results between studies could also be explained by intrinsic differences in individual cohorts and/or different statistical approaches or sample size. Finally, we found significant differences in baseline characteristics of the two groups of patients, including lower FVC %, FEV1 % and 6MWT in the subjects with AISI values over 434, suggesting a more advanced disease in this group.

This study has some limitations, including the relatively small sample size, the retrospective design, and the single-centre nature. Therefore, our findings require confirmation in other populations, ideally in the context of prospective and multicentric studies.

In conclusion, we found that AISI, a simple and inexpensive biomarker, at the time of diagnosis, provides valuable prognostic information and may allow the identification of patients at a higher risk of early mortality. Further prospective studies with larger sample sizes are needed to confirm the usefulness of the AISI to predict mortality in patients with IPF and to guide their management.

## Figures and Tables

**Table 1:** Demographic, clinical, and haematological characteristics of IPF patients.

	IPF (*n* = 82)
Age (years)	72 ± 7
Gender (F/M)	9/73
BMI (kg/m^2^)	26.8 ± 4.0
Smokers (current/former/no)	2/61/19
FEV_1_ (% predicted)	79.1 ± 20.9
FVC (% predicted)	77.0 ± 19.2
TLC (% predicted)	70.0 ± 21.4
DLCO (% predicted)	45.7 ± 18.3
6MWT (meters)	412 ± 138
Disease stage (I/II/III)	33/41/8
Arterial hypertension (yes/no)	43/39
Ischemic cardiopathy or cerebrovascular disease (yes/no %)	16/66
Gastroesophageal reflux disease (yes/no)	25/57
Pulmonary hypertension (yes/no)	16/66
COPD or emphysema (yes/no)	13/69
OSAS (yes/no)	7/74
Antifibrotic drugs (none/Pirf/Nin)	30/46/6
Antiaggregants (yes/no)	33/49
Anticoagulants (yes/no)	10/72
WBC (×10^9^ L)	8.26 (7.21–9.66)
Monocytes (×10^9^ L)	1.83 (1.44–2.29)
Lymphocytes (×10^9^ L)	0.61 (0.50–0.80)
Neutrophils (×10^9^ L)	5.34 (4.36–6.20)
Platelets (×10^9^ L)	237 ± 72
NLR	3.00 (2.25–4.18)
dNLR	1.95 (1.51–2.55)
LMR	3.17 ± 1.36
PLR	118 (93–169)
SII	708 (479-969)
SIRI	1.82 (1.29–2.74)
AISI	434 (282–584)

Data are presented as mean ± standard deviation or median (interquartile range). 6MWT: six-minute walk test; AISI: Aggregate Systemic Inflammation Index; COPD: chronic obstructive pulmonary disease; DLCO: diffusion lung carbon oxide; dNLR: derived neutrophil-to-lymphocyte ratio; FEV1: forced expiratory volume in the 1st second; FVC: forced vital capacity; LMR: lymphocyte-to-monocytes ratio; NLR: neutrophil-to-lymphocyte ratio; OSAS: obstructive sleep apnoea syndrome; PLR: platelet-to-lymphocyte ratio; SII: Systemic Inflammation Index; SIRI: Systemic Inflammation Response Index; TLC: total lung capacity; WBC: white blood cells.

**Table 2:** Relationships between simple blood cell count indexes and clinical parameters.

		DLCO %	FEV1 %	FVC %	TLC %	6MWT	Disease Stage	Survival
WBC	Correlation coefficient Significance level P	−0.340 0.0040	−0.284 0.0117	−0.386 0.0006	−0.394 0.0007	−0.062 0.6114	0.380 0.0017	−0.207 0.0651
Lymphocytes	Correlation coefficient Significance level P	0.094 0.4351	0.001 0.9927	0.005 0.9643	−0.164 0.1684	0.327 0.0057	0.073 0.5588	−0.003 0.9818
Monocytes	Correlation coefficient Significance level P	−0.070 0.5594	−0.096 0.3981	−0.172 0.1376	−0.175 0.1412	−0.082 0.4974	0.100 0.4187	−0.247 0.0265
Neutrophils	Correlation coefficient Significance level P	−0.422 0.0002	−0.332 0.0028	−0.375 0.0008	−0.356 0.0021	−0.176 0.1439	0.324 0.0076	−0.193 0.0850
Platelets	Correlation coefficient Significance level P	−0.041 0.7356	−0.299 0.0075	−0.195 0.0922	−0.231 0.0514	−0.077 0.5286	0.020 0.8708	−0.231 0.0382

6MWT: six-minute walk test; DLCO: diffusion lung carbon oxide; FEV1: forced expiratory volume in the 1st second; FVC: forced vital capacity; TLC: total lung capacity; WBC: white blood cells.

**Table 3:** Relationships between combined blood cell count indexes and clinical parameters.

		DLCO %	FEV1 %	FVC %	TLC %	6MWT	Disease Stage	Survival
NLR	Correlation coefficient Significance level P	−0.373 0.0014	−0.237 0.0358	−0.242 0.0353	−0.106 0.3777	−0.374 0.0014	0.212 0.0843	−0.101 0.3696
dNLR	Correlation coefficient Significance level P	−0.422 0.0003	−0.240 0.0357	−0.198 0.0880	−0.126 0.2978	−0.303 0.0113	0.228 0.0658	−0.083 0.4671
LMR	Correlation coefficient Significance level P	0.148 0.2178	0.055 0.6312	0.106 0.3609	−0.017 0.8897	0.339 0.0041	−0.026 0.8317	0.206 0.0657
PLR	Correlation coefficient Significance level P	−0.128 0.2860	−0.157 0.1663	−0.082 0.4802	−0.001 0.9928	−0.295 0.0133	−0.063 0.6116	−0.188 0.0936
SII	Correlation coefficient Significance level P	−0.365 0.0018	−0.282 0.0117	−0.249 0.0301	−0.200 0.0924	−0.401 0.0006	0.148 0.2328	−0.250 0.0245
SIRI	Correlation coefficient Significance level P	−0.369 0.0015	−0.275 0.0140	−0.337 0.0029	−0.225 0.0573	−0.424 0.0003	0.260 0.0334	−0.278 0.0120
AISI	Correlation coefficient Significance level P	−0.324 0.0055	−0.294 0.0081	−0.324 0.0043	−0.265 0.0234	−0.414 0.0004	0.196 0.1118	−0.344 0.0016

6MWT: six-minute walk test; AISI: Aggregate Systemic Inflammation Index; DLCO: diffusion lung carbon oxide; dNLR: derived neutrophil-to-lymphocyte ratio; FEV_1_: forced expiratory volume in the 1st second; FVC: forced vital capacity; LMR: lymphocyte-to-monocytes ratio; NLR: neutrophil-to-lymphocyte ratio; PLR: platelet-to-lymphocyte ratio; SII: Systemic Inflammation Index; SIRI: Systemic Inflammation Response Index; TLC: total lung capacity.

**Table 4:** Kaplan–Meier survival analysis of simple and combined blood cell count indexes in patients with IPF.

Variable	Median Value	Logrank Test	
		Chi-Squared	*p*-Value
WBC	8.26	2.08	0.15
Lymphocytes	1.83	0.01	0.93
Monocytes	0.61	2.68	0.10
Neutrophils	5.34	0.82	0.36
Platelets	228	1.02	0.31
NLR	3.00	0.31	0.58
dNLR	1.95	0.04	0.84
LMR	3.05	0.26	0.61
PLR	118	0.50	0.48
SII	708	1.60	0.21
SIRI	1.82	1.10	0.29
AISI	434	4.08	0.043

AISI: Aggregate Systemic Inflammation Index; dNLR: derived neutrophil-to-lymphocyte ratio; LMR: lymphocyte-to-monocytes ratio; NLR: neutrophil-to-lymphocyte ratio; PLR: platelet-to-lymphocyte ratio; SII: Systemic Inflammation Index; SIRI: Systemic Inflammation Response Index; WBC: white blood cells.

**Table 5:** Univariate and multivariate Cox regression models showing hazard ratios for the studied variables.

	Univariate		Multivariate	
	HR (95% CI)	*p*-Value	HR (95% CI)	*p*-Value
Age	1.0271 (0.9863–1.0695)	0.20	1.0481 (0.9816–1.1192)	0.16
Gender	0.6803 (0.2439–1.8973)	0.46	0.7934 (0.1812–3.4749)	0.76
BMI	0.8528 (0.7820–0.9299)	0.0003	0.8589 (0.7685–0.9601)	0.007
Smoking status	0.8223 (0.5876–1.1509)	0.25	0.7150 (0.4559–1.1212)	0.14
Disease stage	1.5011 (0.9231–2.4411)	0.10	1.2901 (0.7460–2.2311)	0.36
Antifibrotic drugs	0.7578 (0.4657–1.2330)	0.26	1.0097 (0.5669–1.7981)	0.97
AISI	1.0012 (1.0005–1.0018)	0.0006	1.0017(1.0006–1.0027)	0.003

**Table 6:** Demographic, clinical, and haematological characteristics of IPF patients stratified by AISI status.

	AISI < 434 (*n* = 41)	AISI ≥ 434 (*n* = 41)	*p*-Value
Age (years)	71 ± 6	73 ± 7	0.16
Gender (F/M)	3/38	6/35	0.29
BMI (kg/m^2^)	27.5 ± 4.4	26.1 ± 3.6	0.13
Smokers (current/former/no)	0/33/8	2/28/11	0.23
FEV_1_ (% predicted)	84.7 ± 18.3	73.8 ± 22.0	0.019
FVC (% predicted)	81.6 ± 18.5	72.2 ± 19.1	0.033
TLC (% predicted)	74.4 ± 21.9	65.0 ± 19.9	0.059
DLCO (% predicted)	48.2 ± 15.8	42.9 ± 20.6	0.23
6MWT (meters)	458 ± 116	363 ± 146	0.004
Disease stage (I/II/III)	18/20/3	15/21/5	0.67
Dead/alive	20/21	27/14	0.12
WBC (×10^9^ L)	7.58 (7.01–8.84)	8.59 (7.73–10.87)	0.006
Monocytes (×10^9^ L)	0.56 (0.47–0.72)	0.65 (0.57–0.83)	0.012
Lymphocytes (×10^9^ L)	1.94 (1.70–2.59)	1.56 (1.25–2.17)	0.007
Neutrophils (×10^9^ L)	4.63 (4.08–5.45)	5.74 (5.16–7.34)	<0.001
Platelets (×10^9^ L)	206 ± 60	267 ± 71	<0.001

Data are presented as mean ± standard deviation or median (interquartile range). 6MWT: six-minute walk test; AISI: Aggregate Systemic Inflammation Index; DLCO: diffusion lung carbon oxide; FEV1: forced expiratory volume in the 1st second; FVC: forced vital capacity; TLC: total lung capacity; WBC: white blood cells.

## Data Availability

The datasets used and/or analysed during the current study are available from the corresponding author on reasonable request.

## References

[1] Raghu G., Collard H.R., Egan J.J., Martinez F.J., Behr J., Brown K.K., Colby T.V., Cordier J.F., Flaherty K.R., Lasky J.A. (2011). ATS/ERS/JRS/ALAT Committee on Idiopathic Pulmonary Fibrosis. An official ATS/ERS/JRS/ALAT statement: Idiopathic pulmonary fibrosis: Evidence based guidelines for diagnosis and management. Am. J. Respir. Crit. Care Med..

[2] King T.E., Pardo A., Selman M. (2011). Idiopathic pulmonary fibrosis. Lancet.

[3] Khalil W., Xia H., Bodempudi V., Kahm J., Hergert P., Smith K., Peterson M., Parker M., Herrera J., Bitterman P.B. (2015). Pathologic Regulation of Collagen I by an Aberrant Protein Phos-phatase 2A/Histone Deacetylase C4/MicroRNA-29 Signal Axis in Idiopathic Pulmonary Fibrosis Fibroblasts. Am. J. Respir. Cell. Mol. Biol..

[4] De Vries J., Kessels B.L.J., Drent M. (2001). Quality of life of idiopathic pulmonary fibrosis patients. Eur. Respir. J..

[5] Tomioka H., Imanaka K., Hashimoto K., Iwasaki H. (2007). Health-related Quality of Life in Patients with Idiopathic Pulmonary Fibrosis -Cross-sectional and Longitudinal Study. Intern. Med..

[6] King T.E., Tooze J.A., Schwarz M.I., Brown K.R., Cherniack R.M. (2001). Predicting survival in id-iopathic pulmonary fibrosis: Scoring system and survival model. Am. J. Respir. Crit. Care. Med..

[7] Wells A.U., Desai S.R., Rubens M.B., Goh N.S., Cramer D., Nicholson A.G., Colby T.V., du Bois R.M., Hansell D.M. (2003). Idiopathic pulmonary fibrosis: A composite physiologic index derived from disease extent observed by computed tomography. Am. J. Respir. Crit. Care Med..

[8] Collard H.R., King T.E., Bartelson B.B., Vourlekis J.S., Schwarz M.I., Brown K.K. (2003). Changes in clinical and physiologic variables predict survival in idiopathic pulmonary fibrosis. Am. J. Respir. Crit. Care Med..

[9] Flaherty K.R., Mumford J.A., Murray S., Kazerooni E.A., Gross B.H., Colby T.V., Travis W.D., Flint A., Toews G.B., Lynch J.P. (2003). Prognostic implications of physiologic and radiographic changes in idiopathic interstitial pneumonia. Am. J. Respir. Crit. Care Med..

[10] Nathan S.D., Reffett T., Brown A.W., Fischer C.P., Shlobin O.A., Ahmad S., Weir N., Sheridan M.J. (2013). The Red Cell Distribution Width as a Prognostic Indicator in Idiopathic Pulmonary Fibrosis. Chest.

[11] Desai O., Winkler J., Minasyan M., Herzog E.L. (2018). The Role of Immune and Inflammatory Cells in Idiopathic Pulmonary Fibrosis. Front. Med..

[12] Paliogiannis P., Ginesu G.C., Tanda C., Feo C.F., Fancellu A., Fois A.G., Mangoni A.A., Sotgia S., Carru C., Porcu A. (2018). Inflammatory cell indexes as preoperative predictors of hospital stay in open elective thoracic surgery. ANZ J. Surg..

[13] Paliogiannis P., Satta R., Deligia G., Farina G., Bassu S., Mangoni A.A., Carru C., Zinellu A. (2018). Associations between the neutrophil-to-lymphocyte and the platelet-to-lymphocyte ratios and the presence and severity of psoriasis: A systematic review and meta-analysis. Clin. Exp. Med..

[14] Erre G.L., Paliogiannis P., Castagna F., Mangoni A.A., Carru C., Passiu G., Zinellu A. (2019). Me-ta-analysis of neutrophil-to-lymphocyte and platelet-to-lymphocyte ratio in rheumatoid arthritis. Eur. J. Clin. Investig..

[15] Pinna A., Porcu T., Ricci G.D., Dore S., Boscia F., Paliogiannis P., Carru C., Zinellu A. (2018). Complete Blood Cell Count–Derived Inflammation Biomarkers in Men with Age-Related Macular Degeneration. Ocul. Immunol. Inflamm..

[16] Putzu C., Cortinovis D., Colonese F., Canova S., Carru C., Zinellu A., Paliogiannis P. (2018). Blood cell count indexes as predictors of outcomes in advanced non-small-cell lung cancer patients treated with Nivolumab. Cancer Immunol. Immunother..

[17] Paliogiannis P., Fois A.G., Sotgia S., Mangoni A.A., Zinellu E., Pirina P., Carru C., Zinellu A. (2018). The neutrophil-to-lymphocyte ratio as a marker of chronic obstructive pulmonary disease and its exacerbations: A systematic review and meta-analysis. Eur. J. Clin. Investig..

[18] Paliogiannis P., Fois A.G., Sotgia S., Mangoni A.A., Zinellu E., Pirina P., Negri S., Carru C., Zinellu A. (2018). Neutrophil to lymphocyte ratio and clinical outcomes in COPD: Recent evidence and future perspectives. Eur. Respir. Rev..

[19] Mochimaru T., Ueda S., Suzuki Y., Asano K., Fukunaga K. (2019). Neutrophil-to-lymphocyte ratio as a novel independent predictor of severe exacerbation in patients with asthma. Ann. Allergy Asthma Immunol..

[20] Ruta V., Man A., Alexescu T., Motoc N., Tarmure S., Ungur R., Todea D., Coste S., Valean D., Pop M. (2020). Neutrophil-To-Lymphocyte Ratio and Systemic Immune-Inflammation Index—Biomarkers in Interstitial Lung Disease. Medicina.

[21] Zinellu A., Paliogiannis P., Sotgiu E., Mellino S., Mangoni A.A., Zinellu E., Negri S., Collu C., Pintus G., Serra A. (2020). Blood cell count derived in-flammation indexes in patients with idiopathic pulmonary fibrosis. Lung.

[22] Brusasco V., Crapo R., Viegi G. (2005). Coming together: The ATS/ERS consensus on clinical pulmonary function testing. Eur. Respir. J..

[23] Wynn T.A., Vannella K.M. (2016). Macrophages in Tissue Repair, Regeneration, and Fibrosis. Immunity.

[24] Evans T.C., Jehle D. (1991). The red blood cell distribution width. J. Emerg. Med..

[25] Tsoutsou P.G., Gourgoulianis K.I., Petinaki E., Germenis A., Tsoutsou A.G., Mpaka M., Efremidou S., Molyvdas P.-A. (2006). Cytokine levels in the sera of patients with idiopathic pulmonary fibrosis. Respir. Med..

[26] Ziegenhagen M.W., Zabel P., Zissel G., Schlaak M., Müller-Quernheim J. (1998). Serum level of inter-leukin 8 is elevated in idiopathic pulmonary fibrosis and indicates disease activity. Am. J. Respir. Crit. Care. Med..

[27] Tsoutsou P.G., Gourgoulianis K.I., Petinaki E., Mpaka M., Efremidou S., Maniatis A., Molyvdas P.A. (2004). ICAM-1, ICAM-2 and ICAM-3 in the Sera of Patients with Idiopathic Pulmonary Fibrosis. Inflammation.

[28] Korthagen N.M., van Moorsel C.H., Barlo N.P., Ruven H.J., Kruit A., Heron M., van den Bosch J.M., Grutters J.C. (2011). Serum and BALF YKL-40 levels are predictors of survival in idiopathic pul-monary fibrosis. Respir. Med..

[29] Huszno J., Kolosza Z. (2019). Prognostic value of the neutrophil-lymphocyte, platelet-lymphocyte and monocyte-lymphocyte ratio in breast cancer patients. Oncol. Lett..

[30] Yang A.-P., Liu J.-P., Tao W.-Q., Li H.-M. (2020). The diagnostic and predictive role of NLR, d-NLR and PLR in COVID-19 patients. Int. Immunopharmacol..

[31] Ha R., Lim B.W., Kim D.H., Park J.W., Cho C.H., Lee J.H. (2019). Predictive values of neutrophil to lymphocyte ratio (NLR), platelet to lymphocyte ratio (PLR), and other prognostic factors in pediatric idiopathic sudden sensorineural hearing loss. Int. J. Pediatr. Otorhinolaryngol..

[32] Gao Y.-B., Guo W., Cai S., Zhang F., Shao F., Zhang G., Liu T., Tan F., Li N., Xue Q. (2019). Systemic immune-inflammation index (SII) is useful to predict survival outcomes in patients with surgically resected esophageal squamous cell carcinoma. J. Cancer.

[33] Dong G., Huang A., Liu L. (2020). Platelet-to-lymphocyte ratio and prognosis in STEMI: A meta-analysis. Eur. J. Clin. Investig..

[34] Li Q., Chen P., Shi S., Liu L., Lv J., Zhu L., Zhang H. (2020). Neutrophil-to-lymphocyte ratio as an independent inflammatory indicator of poor prognosis in IgA nephropathy. Int. Immunopharmacol..

[35] Gürün Kaya A., Özyürek B.A., Şahin Özdemirel T., Öz M., Erdoğan Y. (2021). Prognostic Significance of Red Cell Distribution Width in Idiopathic Pulmonary Fibrosis and Combined Pulmonary Fibrosis Emphysema. Med. Princ. Pract..

[36] Bennett D., Salvini M., Fui A., Cillis G., Cameli P., Mazzei M.A., Fossi A., Refini R.M., Rottoli P. (2019). Calgranulin B and KL-6 in Bronchoalveolar Lavage of Patients with IPF and NSIP. Inflammation.

[37] Kreuter M., Lee J.S., Tzouvelekis A., Oldham J.M., Molyneaux P.L., Weycker D., Atwood M., Kirchgaessler K.U., Maher T.M. (2021). Monocyte Count as a Prognostic Biomarker in Patients with Idipathic Pulmonary Fibrosis. Am. J. Respir. Crit. Care Med..

[38] Fois A.G., Paliogiannis P., Scano V., Cau S., Babudieri S., Perra R., Ruzzittu G., Zinellu E., Pirina P., Carru C. (2020). The Sytemic Inflammation Index on Admission Predicts In-Hospital Mortality in COVID-19 Patients. Molecules.

[39] Celli B.R., Cote C.G., Marin J.M., Casanova C., Montes de Oca M., Mendez R.A., Plata V.P., Cabral H.J. (2004). The Body-Mass Index, Airflow Obstruction, Dyspnea, and Exercise Capacity Index in Chronic Obstructive Pulmonary Disease. N. Engl. J. Med..

[40] Prescott E., Almdal T., Mikkelsen K., Tofteng C., Vestbo J., Lange P. (2002). Prognostic value of weight change in chronic obstructive pulmonary disease: Results from the Copenhagen City Heart Study. Eur. Respir. J..

[41] Kulkarni T., Yuan K., Tran-Nguyen T.K., Kim Y.-I., De Andrade J.A., Luckhardt T., Valentine V.G., Kass D.J., Duncan S.R. (2019). Decrements of body mass index are associated with poor outcomes of idiopathic pulmonary fibrosis patients. PLoS ONE.

[42] Alakhras M., Decker P.A., Nadrous H.F., Collazo-Clavell M., Ryu J.H. (2007). Body Mass Index and Mortality in Patients with Idiopathic Pulmonary Fibrosis. Chest.

[43] Nishiyama O., Yamazaki R., Sano H., Iwanaga T., Higashimoto Y., Kume H., Tohda Y. (2016). Fat-free mass index predicts survival in patients with idiopathic pulmonary fibrosis. Respirology.

[44] Zinellu A., Collu C., Zinellu E., Ahmad K., Nasser M., Traclet J., Sotgiu E., Mellino S., Mangoni A.A., Carru C. (2021). IC4: A new combined predictive index of mortality in idiopathic pulmonary fibrosis. Panminerva Med..

[45] Watters L.C., Schwarz M.I., Cherniack R.M., Waldron J.A., Dunn T.L., Stanford R.E., King T.E. (1987). Idiopathic pulmonary fibrosis: Pretreatment bronchoalveolar lavage cellular constituents and their relationships with lung histopathology and clinical response to therapy. Am. Rev. Respir. Dis..

[46] Schwartz D.A., Helmers R.A., Galvin J.R., Van Fossen D.S., Frees K.L., Dayton C.S., Burmeister L.F., Hunninghake G.W. (1994). Determinants of survival in idiopathic pulmonary fibrosis. Am. J. Respir. Crit. Care Med..

[47] Johnston I.D., Prescott R.J., Chalmers J.C., Rudd R.M. (1997). British Thoracic Society study of cryptogenic fibrosing alveolitis: Current presentation and initial management. Fibrosing Alveolitis Subcommittee of the Research Committee of the British Thoracic Society. Thorax.

[48] Baumgartner K.B., Samet J.M., Stidley C.A., Colby T.V., Waldron J.A. (1997). Cigarette smoking: A risk factor for idiopathic pulmonary fibrosis. Am. J. Respir. Crit. Care Med..

[49] Zubairi A.B.S., Ahmad R., Hassan M., Sarwar S., Abbas A., Shahzad T., Irfan M., Muhammad I. (2018). Clinical characteristics and factors associated with mortality in idiopathic pulmonary fibrosis: An experience from a tertiary care center in Pakistan. Clin. Respir. J..

[50] Kishaba T., Nagano H., Nei Y., Yamashiro S. (2016). Clinical characteristics of idiopathic pulmonary fibrosis patients according to their smoking status. J. Thorac. Dis..

[51] King T.E., Schwarz M.I., Brown K., Tooze J.A., Colby T.V., Waldron J.A., Flint A., Thurlbeck W., Cherniack R.M. (2001). Idiopathic pulmonary fibrosis: Relationship between histopathologic features and mortality. Am. J. Respir. Crit. Care Med..

[52] Antoniou K.M., Hansell D.M., Rubens M.B., Marten K., Desai S.R., Siafakas N.M., Nicholson A.G., du Bois R.M., Wells A.U. (2008). Idiopathic pulmonary fibrosis: Outcome in relation to smoking status. Am. J. Respir. Crit. Care Med..

[53] Guenther A., Krauss E., Tello S., Wagner J., Paul B., Kuhn S., Maurer O., Heinemann S., Costabel U., Barbero M.A.N. (2018). The European IPF registry (eurIPFreg): Baseline characteristics and survival of patients with idiopathic pulmonary fi-brosis. Respir. Res..

[54] Behr J., Kreuter M., Hoeper M.M., Wirtz H., Klotsche J., Koschel D., Andreas S., Claussen M., Grohe C., Wilkens H. (2015). Management of patients with idiopathic pulmonary fibrosis in clinical practice: The INSIGHTS-IPF registry. Eur. Respir. J..

[55] Jo H.E., Glaspole I., Grainge C., Goh N., Hopkins P.M., Moodley Y., Reynolds P.N., Chapman S., Walters E.H., Zappala C. (2017). Baseline characteristics of idiopathic pulmonary fibrosis: Analysis from the Australian Idiopathic Pulmonary Fibrosis Registry. Eur. Respir. J..

[56] Richeldi L., du Bois R.M., Raghu G., Azuma A., Brown K.K., Costabel U., Cottin V., Flaherty K.R., Hansell D.M., Inoue Y. (2014). Efficacy and safety of nintedanib in idiopathic pul-monary fibrosis. N. Engl. J. Med..

[57] King T.E., Bradford W.Z., Castro-Bernardini S., Fagan E.A., Glaspole I., Glassberg M.K., Gorina E., Hopkins P.M., Kardatzke D., Lancaster L. (2014). A phase 3 trial of pirfenidone in patients with idiopathic pulmonary fibrosis. N. Engl. J. Med..

[58] Kolb M., Raghu G., Wells A.U., Behr J., Richeldi L., Schinzel B., Quaresma M., Stowasser S., Martinez F.J., INSTAGE Investigators (2018). Nintedanib plus Sildenafil in Patients with Idiopathic Pul-monary Fibrosis. N. Engl. J. Med..

[59] Sesé L., Nunes H., Cottin V., Israel-Biet D., Crestani B., Guillot-Dudoret S., Cadranel J., Wal-laert B., Tazi A., Maître B. (2021). Gender Differences in Idiopathic Pulmonary Fibrosis: Are Men and Women Equal?. Front. Med..

[60] Durheim M.T., Judy J., Bender S., Neely M.L., Baumer D., Robinson S.B., Conoscenti C.S., Leonard T.B., Lazarus H.M., Palmer S.M. (2020). A retrospective study of in-hospital mortality in patients with idiopathic pulmonary fibrosis between 2015 and 2018. Medicine.

[61] Marcon A., Schievano E., Fedeli U. (2021). Mortality Associated with Idiopathic Pulmonary Fibrosis in Northeastern Italy, 2008–2020: A Multiple Cause of Death Analysis. Int. J. Environ. Res. Public Health.

[62] Kim H.J., Perlman D., Tomic R. (2015). Natural history of idiopathic pulmonary fibrosis. Respir. Med..

[63] López-Muñiz Ballesteros B., López-Herranz M., Lopez-de-Andrés A., Hernandez-Barrera V., Jiménez-García R., Carabantes-Alarcon D., Jiménez-Trujillo I., de Miguel-Diez J. (2021). Sex Differ-ences in the Incidence and Outcomes of Patients Hospitalized by Idiopathic Pulmonary Fibrosis (IPF) in Spain from 2016 to 2019. J. Clin. Med..

[64] Nadrous H.F., Ryu J.H., Douglas W.W., Decker P.A., Olson E.J. (2004). Impact of Angiotensin-Converting Enzyme Inhibitors and Statins on Survival in Idiopathic Pulmonary Fibrosis. Chest.

[65] Song H., Sun D., Ban C., Liu Y., Zhu M., Ye Q., Yan W., Ren Y., Dai H. (2019). Independent Clinical Factors Relevant to Prognosis of Patients with Idiopathic Pulmonary Fibrosis. Med. Sci. Monit..

